# Revisiting Why Plants Become N Deficient Under Elevated CO_2_: Importance to Meet N Demand Regardless of the Fed-Form

**DOI:** 10.3389/fpls.2021.726186

**Published:** 2021-11-04

**Authors:** Maaya Igarashi, Yan Yi, Katsuya Yano

**Affiliations:** Laboratory of Crop Science, Graduate School of Bioagricultural Sciences, Nagoya University, Nagoya, Japan

**Keywords:** ammonium, cumulative transpiration, nitrate, nitrogen nutrition, water-use efficiency

## Abstract

An increase in plant biomass under elevated CO_2_ (eCO_2_) is usually lower than expected. N-deficiency induced by eCO_2_ is often considered to be a reason for this. Several hypotheses explain the induced N-deficiency: (1) eCO_2_ inhibits nitrate assimilation, (2) eCO_2_ lowers nitrate acquisition due to reduced transpiration, or (3) eCO_2_ reduces plant N concentration with increased biomass. We tested them using C_3_ (wheat, rice, and potato) and C_4_ plants (guinea grass, and *Amaranthus*) grown in chambers at 400 (ambient CO_2_, aCO_2_) or 800 (eCO_2_) μL L^−1^ CO_2_. In most species, we could not confirm hypothesis (1) with the measurements of plant nitrate accumulation in each organ. The exception was rice showing a slight inhibition of nitrate assimilation at eCO_2_, but the biomass was similar between the nitrate and urea-fed plants. Contrary to hypothesis (2), eCO_2_ did not decrease plant nitrate acquisition despite reduced transpiration because of enhanced nitrate acquisition per unit transpiration in all species. Comparing to aCO_2_, eCO_2_ remarkably enhanced water-use efficiency, especially in C_3_ plants, decreasing water demand for CO_2_ acquisition. As our results supported hypothesis (3) without any exception, we then examined if lowered N concentration at eCO_2_ indeed limits the growth using C_3_ wheat and C_4_ guinea grass under various levels of nitrate-N supply. While eCO_2_ significantly increased relative growth rate (RGR) in wheat but not in guinea grass, each species increased RGR with higher N supply and then reached a maximum as no longer N was limited. To achieve the maximum RGR, wheat required a 1.3-fold N supply at eCO_2_ than aCO_2_ with 2.2-fold biomass. However, the N requirement by guinea grass was less affected by the eCO_2_ treatment. The results reveal that accelerated RGR by eCO_2_ could create a demand for more N, especially in the leaf sheath rather than the leaf blade in wheat, causing N-limitation unless the additional N was supplied. We concluded that eCO_2_ amplifies N-limitation due to accelerated growth rate rather than inhibited nitrate assimilation or acquisition. Our results suggest that plant growth under higher CO_2_ will become more dependent on N but less dependent on water to acquire both CO_2_ and N.

## Introduction

Approximately 90% of plant dry matter consists of C and O (Epstein and Bloom, 2005), mainly derived from atmospheric CO_2_. Higher atmospheric CO_2_ concentrations have the potential to increase plant biomass because (1) CO_2_ is the substrate for photosynthesis in plants, and (2) the photosynthetic rate is not yet saturated under the current ambient CO_2_ concentration (aCO_2_), particularly in C_3_ plants (Lemonnier and Ainsworth, 2018). However, plant growth enhancement under elevated CO_2_ (eCO_2_) is almost always lower than expected (Kimball et al., 1993; Ainsworth and Long, 2005). It is frequently pointed out that the reason for this growth shortness is that plants under eCO_2_ suffer from N-deficiency. Hence the growth is more limited by N compared with aCO_2_ treatments (Poorter et al., 1997; Cotrufo et al., 1998; Gifford et al., 2000; Taub and Wang, 2008; Feng et al., 2015). To fully realize the effects of CO_2_ fertilization, such eCO_2_-induced N-limitation must be overcome. Therefore, it is critical to clarify why plants are more prone to N deficiency under eCO_2_ treatments (Ainsworth and Long, 2005).

Here, we tested three hypotheses to elucidate the cause of eCO_2_-induced N-limitation: (1) eCO_2_ may inhibit the reduction of ${\text{NO}}_{3}^{-}$ to ${\text{NH}}_{4}^{+}$ by the shortage of reductants, such as NADH, with lower photorespiration, resulting in nitrate accumulation instead of organic-N shortage in plant tissues (Rachmilevitch et al., 2004; Bloom et al., 2010, 2012; Rubio-Asensio et al., 2015); (2) eCO_2_ may decrease nitrate acquisition *via* reduced transpiration with lower stomatal conductance as transpiration is the main driving-force for ${\text{NO}}_{3}^{-}$ movement in the soil (Conroy, 1992; Taub and Wang, 2008; McGrath and Lobell, 2013; Feng et al., 2015); and (3) stimulation of photosynthesis under eCO_2_ may directly increase carbohydrate production, and thus, the N concentration in the tissue may decrease as a growth dilution effect unless N acquisition by the plant increases accordingly (Poorter et al., 1997; Gifford et al., 2000; Taub and Wang, 2008).

If hypotheses (1) is responsible for the eCO_2_-induced N-limitation, partially feeding with ${\text{NH}}_{4}^{+}$ instead of ${\text{NO}}_{3}^{-}$ may alleviate it because of less reductant requirement. Hypothesis (2) is also true when the N source for plants is ${\text{NO}}_{3}^{-}$ because its movement in soil is highly dependent on transpiration-driven mass flow. Taub and Wang (2008) pointed out that the decrease in concentration under elevated CO_2_ is the highest for macronutrients that are supplied to the roots by transpiration-driven mass flow (nitrate-N, Mg, and Ca) and it is the least for those most dependent on diffusion through the soil (P and K). Therefore, feeding with ${\text{NH}}_{4}^{+}$, which is similar to K^+^ in the soil, may allow plant N acquisition to be less affected by lowered transpiration. On the contrary, when hypothesis (3) can explain the N-limitation, an important issue is whether lowered N concentration at eCO_2_ actually limits the growth or not.

To examine these hypotheses, we compared soil-grown plants fed with nitrate or urea, which releases ${\text{NH}}_{4}^{+}$ in the soil environment. Because ${\text{NH}}_{4}^{+}$ is readily oxidized to ${\text{NO}}_{3}^{-}$ by soil microbes, urea and nitrate were applied weekly to maintain fresh ${\text{NH}}_{4}^{+}$ released from it. This was not intended to completely control ${\text{NO}}_{3}^{-}$ or ${\text{NH}}_{4}^{+}$ as the sole N source as in hydroponics, but to provide reduced-N in addition to ${\text{NO}}_{3}^{-}$ for application to field crops grown in soil. Further, we used various monocotyledonous (wheat, rice, and guinea grass) and dicotyledonous (potato and *Amaranthus*) plants that employ C_3_ (wheat, rice, and potato) or C_4_ (guinea grass and *Amaranthus*) photosynthesis mechanisms to examine whether the effects of eCO_2_ on nitrate assimilation and acquisition differ between C_3_ or C_4_ plants. This is because C_4_ plants have inherently less photorespiration and relatively smaller stomatal openings compared with C_3_ plants (Imai and Okamoto-Sato, 1991; Ward et al., 1999; Cousins and Bloom, 2003; Lambers et al., 2008). Using the suitable N-form based on the obtained results, we further quantified the growth responses of wheat and guinea grass as representatives of C_3_ and C_4_ plants, respectively, against N supply at each CO_2_ value. To date, such attempts have been rarely made, as most studies have assessed the qualitative results of high contrasts (e.g., high N vs. low N).

Here, we attempted to answer the following questions:

1. Does eCO_2_ inhibit nitrate assimilation or nitrate acquisition, or both?
2. Is the growth of plants fed with reduced-N (i.e., urea) greater than those fed with nitrate under eCO_2_?
3. What is the quantity of N supply that is required for maximum plant growth at eCO_2_?

In addition, we paid special attention to the water-use efficiency (WUE) at the individual plant level (i.e., biomass production per transpiration). This is because an increase in plant biomass is more likely responsible for WUE rather than the amount of water transpired under eCO_2_ (Yi et al., 2019, 2020; Yi and Yano, 2021), which hardly occurs under current aCO_2_ treatments except an improvement in nocturnal transpiration (Coupel-Ledru et al., 2016). In this study, we aimed to explore how to improve eCO_2_-induced N-limitation by answering the above questions.

## Materials and Methods

### Plant Growth (Experiment 1)

Wheat (*Triticum aestivum* L. “Ayahikari”), rice (*Oryza sativa* L. “Nipponbare”), and potato (*Solanum tuberosum* L. “Irish Cobbler”) were selected to represent C_3_ plants, and guinea grass (*Panicum maximum* Jacq. “Natsukaze”) and *Amaranthus* spp. (Tusrushin seeds, Co., Ltd., Japan) were selected to represent C_4_ plants. Seeds were sown into trays filled with vermiculite and grown in controlled environment chambers (LPH-410 SPC, Nippon Medical and Chemical Instruments Co., Ltd., Japan) with the following conditions: light intensity, 400 μmol m^−2^ s^−1^; relative humidity, 60%; temperature, 30/25°C (day/night); and photoperiod, 14/10 h (day/night). Potato tubers were cut into ~6.45 g pieces, buried in the tray, and sprouted in a controlled-environment room with the following conditions: light intensity, 150 μmol m^−2^ s^−1^; relative humidity, 70%; temperature, 24/24°C (day/night); and photoperiod, 12/12 h (day/night). After sprouting to ~5 cm in length, the tuber pieces were placed in the same chamber as the seedlings of the other species. Before transplanting, each seedling received 21 ml of a nutrient solution (Hyponex liquid fertilizer, Hyponex Japan Co., Ltd., Japan) diluted at 1/1,000 with tap water, and sprouted potato tuber pieces received 25 mL of the nutrient solution diluted at 1/500 with tap water.

The seedlings of each species were then transplanted into 1-L pots (11.3 × 14.0 cm, diameter × depth; one plant per pot) without holes for drainage, and were filled with 643 g of dry Andosol, in which 0.32 g of potassium chloride (60.0% K_2_O) and 5.05 g of calcium superphosphate (17.5% P_2_O_5_) were uniformly mixed per pot. N was applied weekly using aliquots of 1 M NaNO_3_ or 1 M urea diluted with distilled water to achieve a final N content of 0.19 g per pot, 0.03 g of N at transplanting, 0.03 g of N at 7 d after transplanting (DAT), 0.05g of N at 14 DAT, and 0.08 g of N at 21 DAT. The split application was intended to supply weekly fresh ammonium ions released from urea as previously supplied ones were readily oxidized to nitrate in the soil. We observed that the half-life of ammonium-N was ~10 d in the moistened soil without plants when urea was applied. Each plant was grown using two chambers [light intensity, 400 μmol m^−2^ s^−1^; relative humidity, 60%; temperature, 27/17°C (day/night); and photoperiod, 12/12 h (day/night) at ~400 μL L^−1^ for aCO_2_ and 800 μL L^−1^ for eCO_2_]. The actual CO_2_ concentration (mean ± SE during the growth period) monitored in each chamber was 397 ± 9 μL L^−1^ (day) and 569 ± 12 μL L^−1^ (night) under aCO_2_, and 749 ± 10 μL L^−1^ (day) and 711 ± 11 μL L^−1^ (night) under eCO_2_. The plants and CO_2_ concentrations were switched weekly between the two chambers to minimize any potential chamber effects. Each plant was grown for 28 d and then harvested.

Sampling was conducted twice, at transplanting and harvesting, to conduct growth analysis (Hunt et al., 2002), in which relative growth rate (RGR), net assimilation rate (NAR), and leaf area ratio (LAR) were calculated using the following equations (Saeki, 1965):


$$\begin{array}{l} \text{RGR}({\text{g g}}^{-\text{1}}{\text{d}}^{-\text{1}})=({\text{log}}_{\text{e}}W_{2}-{\text{log}}_{\text{e}}W_{1})/(t_{2}-\;t_{1}) \end{array}$$

$$\begin{array}{l} \text{NAR}({\text{g m}}^{-\text{2}}{\text{d}}^{-\text{1}})=[(W_{2}-W_{1})/(t_{2}-t_{1})]\times \\ \;[({\text{log}}_{\text{e}}LA_{2}-{\text{log}}_{\text{e}}LA_{1})/(LA_{2}-\;LA_{1})] \end{array}$$

$$\begin{array}{l} \text{LAR}({\text{m}}^{2}{\text{g}}^{-\text{1}})=[({\text{log}}_{\text{e}}W_{2}-{\text{log}}_{\text{e}}W_{1})/(W_{2}-W_{1})]\times \\ \;[(LA_{2}-LA_{1})/({\text{log}}_{\text{e}}LA_{2}-{\text{log}}_{\text{e}}\;LA_{1})] \end{array}$$

where, *W*_1_: dry weight at transplanting, *W*_2_: dry weight at harvesting, *LA*_1_: leaf area at transplanting, *LA*_2_: leaf area at harvesting, *t*_1_: day of transplanting, and *t*_2_: day of harvesting. Leaf area and root length were measured immediately after sampling using WinRHIZO Pro LA2400 (Regent Instruments Inc., Canada) before drying. The leaves, stems or leaf sheaths, and roots were separately dried in an oven at 80°C for 48 h and then weighed. After drying, each plant part was separately ground to powder for chemical analysis.

### Plant Growth (Experiment 2)

Wheat (*T. aestivum* L. “Ayahikari”) and guinea grass (*P. maximum* Jacq. “Natsukaze”) were used. The growth conditions were the same as those described in Experiment 1, except for the N-fertilizer application. Using 1 M NaNO_3_ solution, 8 levels of N supply rates were prepared at transplanting (0, 0.02, 0.03, 0.06, 0.13, 0.19, 0.26, and 0.32 g N per pot). Each plant was grown in controlled environment chambers with the same conditions and growth periods used in Experiment 1. The actual CO_2_ concentration (mean ± SE during the growth period) monitored in each chamber was 402 ± 14 μL L^−1^ (day) and 526 ± 72 μL L^−1^ (night) under aCO_2_, and 831 ± 8 μL L^−1^ (day) and 789 ± 7 μL L^−1^ (night) under eCO_2_.

### Measurement of Cumulative Transpiration

Immediately after transplanting, each pot received 417 mL of tap water to achieve 65% (v/w) of the initial soil water content. Following a previously described method (Yi et al., 2019, 2020), the daily water consumption was measured by weighing each pot covered with a transparent vinyl sheet to prevent evaporation, and then tap water was supplied to maintain the initial soil water content. The cumulative transpiration throughout the growth period was calculated in each pot using the water consumption that was recorded daily. The WUE was calculated as the total plant biomass/cumulative transpiration (Jones, 2004).

### Measurements of the Total C, Total N, Nitrate-N Concentrations, and ^15^N in Plants

Dried and ground samples were simultaneously analyzed to determine the total C and N concentrations using an elemental analyzer (NA2500; CE Instruments, Milan, Italy). To determine the nitrate concentration in the tissues, samples of ~50 mg were extracted in 5 mL of distilled water in a hot bath at 100°C for 30 min and then centrifugated at 2600 g for 2 min. The nitrate concentration in the supernatant was colorimetrically determined according to Cataldo et al. (1975). The precipitate was collected and re-dried in an oven at 80°C for 48 h to measure the δ^15^N ratio. The dried precipitate from each plant part was thoroughly mixed based on the weight ratio of each part. The mixed sample for each plant was combusted in an elemental analyzer (NA2500; CE Instruments, Milan, Italy). A part of the combustion gases was introduced into an isotopic ratio mass spectrometer (Delta Plus, Thermo Fisher Scientific Inc. Worcester, MA, USA), and the δ^15^N value was determined.

### Statistical Analysis

Experiment 1 was organized following a factorial design with two CO_2_ concentrations, two N-forms, and five plant species with four biological replicates. The data were analyzed using a two-way analysis of variance (ANOVA), in which the sources of variance were CO_2_ concentration (aCO_2_ or eCO_2_), N forms (nitrate or urea), and their interactions within each species. Experiment 2 consisted of two CO_2_ concentrations, two plant species, and eight levels of N supply rates, and compared the growth responses of each species against N supply under each CO_2_ treatment. In Experiment 2, there were no biological replicates as our intention was to compare growth responses but not means. In such a case, replicating observations is a necessary sense and loses sensitivity (Barrow, 2021). The main effects of N supply and CO_2_ treatment within each species were analyzed using a two-way ANOVA without replication.

## Results

### Experiment 1

The RGR was not affected by the form of N fertilizer in wheat, rice, potato, and guinea grass not only under eCO_2_ but also under aCO_2_ (Table 1) without any visible symptoms, implying that toxic effects of ammonia released from urea were not detectable. As for these species, the RGR was higher under eCO_2_ than under aCO_2_. The only exception was *Amaranthus*, which showed a higher RGR under the nitrate-fed treatment than under the urea-fed treatment, while the effect of the CO_2_ level was not significant (Table 1). The increase in the RGR by CO_2_ enrichment was higher in C_3_ plants (10–20% increase) than in C_4_ guinea grass (~3% increase).

**Table 1:** Growth parameters of nitrate-fed or urea-fed plants in five species grown for 28 d in the chambers under ambient (aCO_2_) or elevated (eCO_2_) CO_2_ treatments.

**Species**	**CO_**2**_**	**N-fed form**	**Relative growth rate** **(g g^**−1**^ day^**−1**^)**	**Transpiration** **(L plant^**−1**^)**	**Water-use efficiency** **(g L^**−1**^)**	**N acquisition** **(mg N plant^**−1**^)**	**N acquisition per transpiration** **(mg N L^**−1**^)**
Wheat	aCO_2_	Nitrate	0.144 ± 0.002	1.67 ± 0.06	4.12 ± 0.16	204 ± 1	123 ± 4
		Urea	0.147 ± 0.005	1.56 ± 0.08	4.82 ± 0.45	196 ± 8	126 ± 3
	eCO_2_	Nitrate	0.158 ± 0.005	1.32 ± 0.10	7.87 ± 0.51	209 ± 6	161 ± 10
		Urea	0.157 ± 0.003	1.33 ± 0.05	7.54 ± 0.42	217 ± 2	164 ± 7
	**ANOVA**	**CO**_**2**_ **(C)**	***P*** **=** **0.016**	***P*** **=** **0.006**	***P*** **<** **0.001**	***P*** **=** **0.044**	***P*** **<** **0.001**
		**N form (N)**	***P*** **=** **0.843**	***P*** **=** **0.582**	***P*** **=** **0.703**	***P*** **=** **0.947**	***P*** **=** **0.670**
		**C** **×** **N**	***P*** **=** **0.718**	***P*** **=** **0.473**	***P*** **=** **0.296**	***P*** **=** **0.204**	***P*** **=** **0.929**
Rice	aCO_2_	Nitrate	0.147 ± 0.003	0.48 ± 0.03	3.93 ± 0.07	64 ± 4	132 ± 2
		Urea	0.145 ± 0.004	0.46 ± 0.04	3.91 ± 0.04	65 ± 6	141 ± 2
	eCO_2_	Nitrate	0.161 ± 0.003	0.48 ± 0.03	5.79 ± 0.22	84 ± 5	173 ± 7
		Urea	0.168 ± 0.003	0.56 ± 0.05	6.10 ± 0.19	100 ± 9	178 ± 2
	**ANOVA**	**CO**_**2**_ **(C)**	***P*** **<** **0.001**	***P*** **=** **0.256**	***P*** **<** **0.001**	***P*** **=** **0.002**	***P*** **<** **0.001**
		**N form (N)**	***P*** **=** **0.512**	***P*** **=** **0.522**	***P*** **=** **0.432**	***P*** **=** **0.226**	***P*** **=** **0.145**
		**C** **×** **N**	***P*** **=** **0.240**	***P*** **=** **0.293**	***P*** **=** **0.372**	***P*** **=** **0.297**	***P*** **=** **0.654**
Potato	aCO_2_	Nitrate	0.046 ± 0.002	1.23 ± 0.04	2.98 ± 0.19	206 ± 10	167 ± 7
		Urea	0.052 ± 0.001	1.32 ± 0.03	3.52 ± 0.10	211 ± 3	160 ± 1
	eCO_2_	Nitrate	0.056 ± 0.003	1.18 ± 0.03	4.53 ± 0.37	217 ± 3	184 ± 5
		Urea	0.058 ± 0.002	1.19 ± 0.03	4.84 ± 0.25	208 ± 2	174 ± 4
	**ANOVA**	**CO**_**2**_ **(C)**	***P*** **=** **0.006**	***P*** **=** **0.025**	***P*** **<** **0.001**	***P*** **=** **0.559**	***P*** **=** **0.013**
		**N form (N)**	***P*** **=** **0.097**	***P*** **=** **0.177**	***P*** **=** **0.159**	***P*** **=** **0.739**	***P*** **=** **0.138**
		**C** **×** **N**	***P*** **=** **0.437**	***P*** **=** **0.320**	***P*** **=** **0.702**	***P*** **=** **0.276**	***P*** **=** **0.799**
							
Guinea grass	aCO_2_	Nitrate	0.221 ± 0.002	1.14 ± 0.97	9.94 ± 0.29	204 ± 3	179 ± 6
		Urea	0.221 ± 0.001	1.12 ± 0.02	10.04 ± 0.20	211 ± 2	189 ± 3
	eCO_2_	Nitrate	0.228 ± 0.001	0.97 ± 0.02	13.94 ± 0.25	219 ± 4	227 ± 7
		Urea	0.226 ± 0.002	0.94 ± 0.03	13.66 ± 0.19	208 ± 6	223 ± 5
	**ANOVA**	**CO**_**2**_ **(C)**	***P*** **=** **0.004**	***P*** **<** **0.001**	***P*** **<** **0.001**	***P*** **=** **0.210**	***P*** **<** **0.001**
		**N form (N)**	***P*** **=** **0.456**	***P*** **=** **0.379**	***P*** **=** **0.738**	***P*** **=** **0.643**	***P*** **=** **0.661**
		**C** **×** **N**	***P*** **=** **0.617**	***P*** **=** **0.902**	***P*** **=** **0.504**	***P*** **=** **0.081**	***P*** **=** **0.306**
*Amaranthus*	aCO_2_	Nitrate	0.174 ± 0.001	1.26 ± 0.04	5.91 ± 0.14	248 ± 2	196 ± 6
		Urea	0.165 ± 0.003	1.17 ± 0.04	5.03 ± 0.21	247 ± 5	211 ± 10
	eCO_2_	Nitrate	0.173 ± 0.004	0.96 ± 0.11	7.72 ± 0.21	244 ± 9	267 ± 31
		Urea	0.167 ± 0.001	0.93 ± 0.03	6.53 ± 0.21	247 ± 3	2641 ± 7
	**ANOVA**	**CO**_**2**_ **(C)**	***P*** **=** **0.989**	***P*** **=** **0.004**	***P*** **<** **0.001**	***P*** **=** **0.781**	***P*** **=** **0.008**
		**N form (N)**	***P*** **=** **0.036**	***P*** **=** **0.446**	***P*** **=** **0.001**	***P*** **=** **0.873**	***P*** **=** **0.761**
		**C** **×** **N**	***P*** **=** **0.664**	***P*** **=** **0.678**	***P*** **=** **0.500**	***P*** **=** **0.725**	***P*** **=** **0.660**

*Each data is mean ± SE (n = 4). The bold values indicate probabilities by two-way analysis of variance (ANOVA)*.

Water consumption (i.e., cumulative transpiration during the 28-d experimental period) was lower under eCO_2_ than under aCO_2_ in most species except rice (Table 1). Rice increased both leaf area and root length but decreased leaf area per root length with CO_2_ enrichment, which was not observed in the other species (Supplementary Table 1). The WUE was remarkably enhanced under the eCO_2_ treatment in all species (*p* < 0.001), with the highest increase observed in nitrate-fed wheat (1.9-fold) and the lowest increase in nitrate-fed *Amaranthus* (1.1-fold). However, the form of N fertilizer did not significantly affect the WUE, except for *Amaranthus* (Table 1).

The amount of N acquired throughout the 28 d was calculated by subtracting the plant N content at transplanting from that at sampling in each species. The eCO_2_ treatment enhanced the N acquisition in wheat and rice but not in potato, guinea grass, and *Amaranthus* (Table 1). In all species, CO_2_ enrichment significantly increased the N acquisition per unit transpiration (Table 1). Changes in the leaf area and the root length, including the ratio, with CO_2_ enrichment (Supplementary Table 1) did not correspond to such consistent increases in the N acquisition per transpiration across the species.

**Figure 1:**
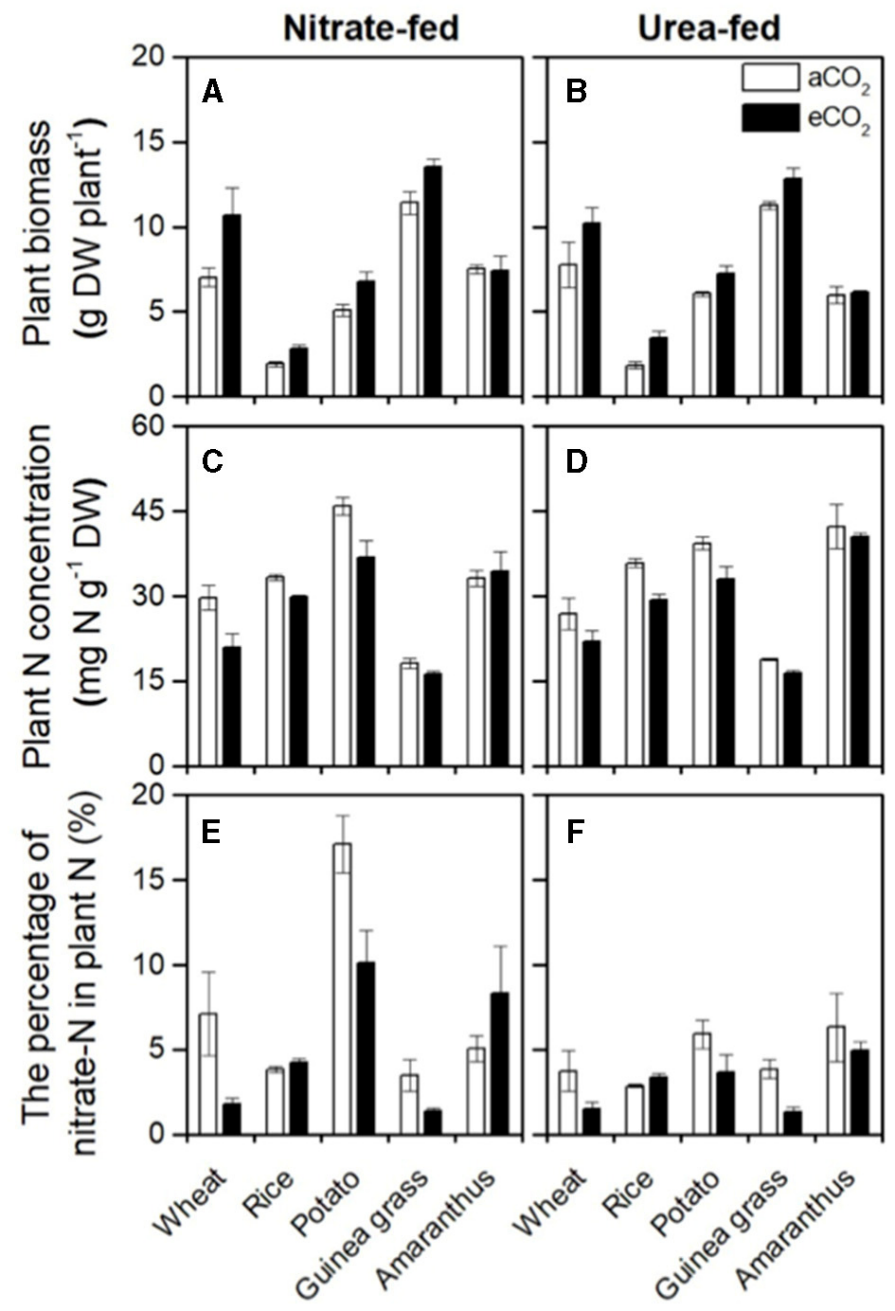
Plant biomass **(A,B)**, plant N concentration **(C,D)**, and the percentage of nitrate-N in total N **(E,F)** in nitrate-fed or urea-fed plants in five species grown for 28 d in the chambers under ambient (aCO_2_) or elevated (eCO_2_) CO_2_ treatments.

According to the RGR, eCO_2_ increased the plant biomass of the sampled plants (Figures 1A,B), although the form of N fertilizer did not significantly affect the biomass of each species, except *Amaranthus* (Table 2). As a result, 1.4, 1.7, 1.3, and 1.2-fold increases in biomass were observed in wheat, rice, potato, and guinea grass plants, respectively. The amount of biomass in each organ is shown in Supplementary Table 2.

**Table 2:** Probabilities by two-way analysis of variance (ANOVA) for plant biomass, plant N concentration, and the percentage of nitrate-N in plant total N in nitrate-fed or urea-fed plants in five species grown for 28 d in the chambers under ambient (aCO_2_) or elevated (eCO_2_) CO_2_ treatments.

**Species**	**Source of variance**	**Plant biomass (g DW plant^**−1**^)**	**Plant N conc. (mg N g^**−1**^DW)**	**Nitrate-N in plant N (%)**
Wheat	CO_2_ (C)	0.026	0.015	0.019
	N form (N)	0.903	0.730	0.209
	C × N	0.631	0.440	0.292
Rice	CO_2_ (C)	<0.001	<0.001	0.040
	N form (N)	0.310	0.243	<0.001
	C × N	0.188	0.075	0.835
Potato	CO_2_ (C)	0.006	0.004	0.008
	N form (N)	0.129	0.036	<0.001
	C × N	0.556	0.550	0.131
Guinea grass	CO_2_ (C)	0.005	0.003	0.002
	N form (N)	0.428	0.517	0.779
	C × N	0.621	0.582	0.696
*Amaranthus*	CO_2_ (C)	0.963	0.566	0.615
	N form (N)	0.023	0.004	0.560
	C × N	0.811	0.825	0.222

Although the foliar N concentration on the area basis was not affected by either CO_2_ or the form of N fertilizer in each species (Supplementary Figures 1A,B), the plant N concentration on the mass basis (Figures 1C,D), the total N (Supplementary Table 3), and organic-N (Supplementary Table 4) in each organ was considerably decreased under the eCO_2_ treatment in all species except *Amaranthus*, in which biomass was not affected by CO_2_ (Table 2). In addition, the plant N concentration was also significantly affected by the form of N fertilizer in potato and *Amaranthus* (Table 2), where the potato had a higher plant N concentration in the nitrate-fed treatment (with a ~three-fold increase in the nitrate-N percentage as shown in Figures 1E,F). In contrast, *Amaranthus* showed a higher N concentration under the urea-fed treatment (Figures 1C,D). However, we did not observe any differences due to the treatments in the foliar N concentration on the area basis within each species (Supplementary Figures 1A,B). The leaf mass per area was significantly increased by CO_2_ enrichment in each species, except in *Amaranthus* again (Supplementary Figures 1C,D).

The percentage of nitrate-N in total plant N was investigated to evaluate nitrate accumulation (Figures 1E,F). Urea-fed plants had a certain amount of nitrate-N (Figure 1F) due to nitrate recently oxidized from ammonium in addition to the initial amount in the soil. Nitrate-fed plants showed a relatively higher percentage than urea-fed plants, especially in the C_3_ plants (Figure 1E), but it was species dependent. A significant increase in the percentage was detected in rice and potato but not in the other species (Table 2). The percentage was significantly affected by CO_2_ enrichment in each species, except in *Amaranthus* (Table 2). However, CO_2_ enrichment could increase the percentage only in rice, and other species (wheat, potato, and rice) showed a decrease in the percentage under eCO_2_ compared with that under aCO_2_ (Figures 1E,F). In each species, CO_2_ enrichment could affect the percentage of nitrate-N in the shoots but not in the roots (Supplementary Table 5).

To further confirm the effect of eCO_2_ on nitrate assimilation, we investigated the δ^15^N in the residues of the plant samples after nitrate extraction. We assumed that N in the residues would approximately reflect plant organic-N, although some contamination of residual nitrate-N and removal of water-soluble organic-N might also be involved. In principle, when nitrate reductase activity (i.e., demand) is relatively lower than the amount of available substrate (i.e., supply), the enzyme preferentially catalyzes ^14^${\text{NO}}_{3}^{-}$ over ^15^${\text{NO}}_{3}^{-}$, resulting in a lower ^15^N/^14^N ratio in plant organic-N (lower δ^15^N value). Thus, the δ^15^N value in plant organic-N was expected to decrease when nitrate reductase activity was inhibited under the eCO_2_ treatment. However, all species, except rice, did not show a decrease in the δ^15^N values in the residues under eCO_2_ compared with those observed under aCO_2_ (Supplementary Table 5). Only rice indicated a lower δ^15^N value in the residues along with nitrate accumulation promoted by CO_2_ enrichment.

### Experiment 2

As the data in Experiment 1 indicated that CO_2_ enrichment did not necessarily inhibit nitrate assimilation and N acquisition but decreased the plant N concentration on a mass basis, we examined whether an increase in the N supply could improve plant growth while at the same time prevent N deficiency under CO_2_ enrichment. The growth response to the nitrate-N supply level was investigated using C_3_ wheat and C_4_ guinea grass to determine the quantity of N that is required for maximum growth in each CO_2_ treatment.

**Figure 2:**
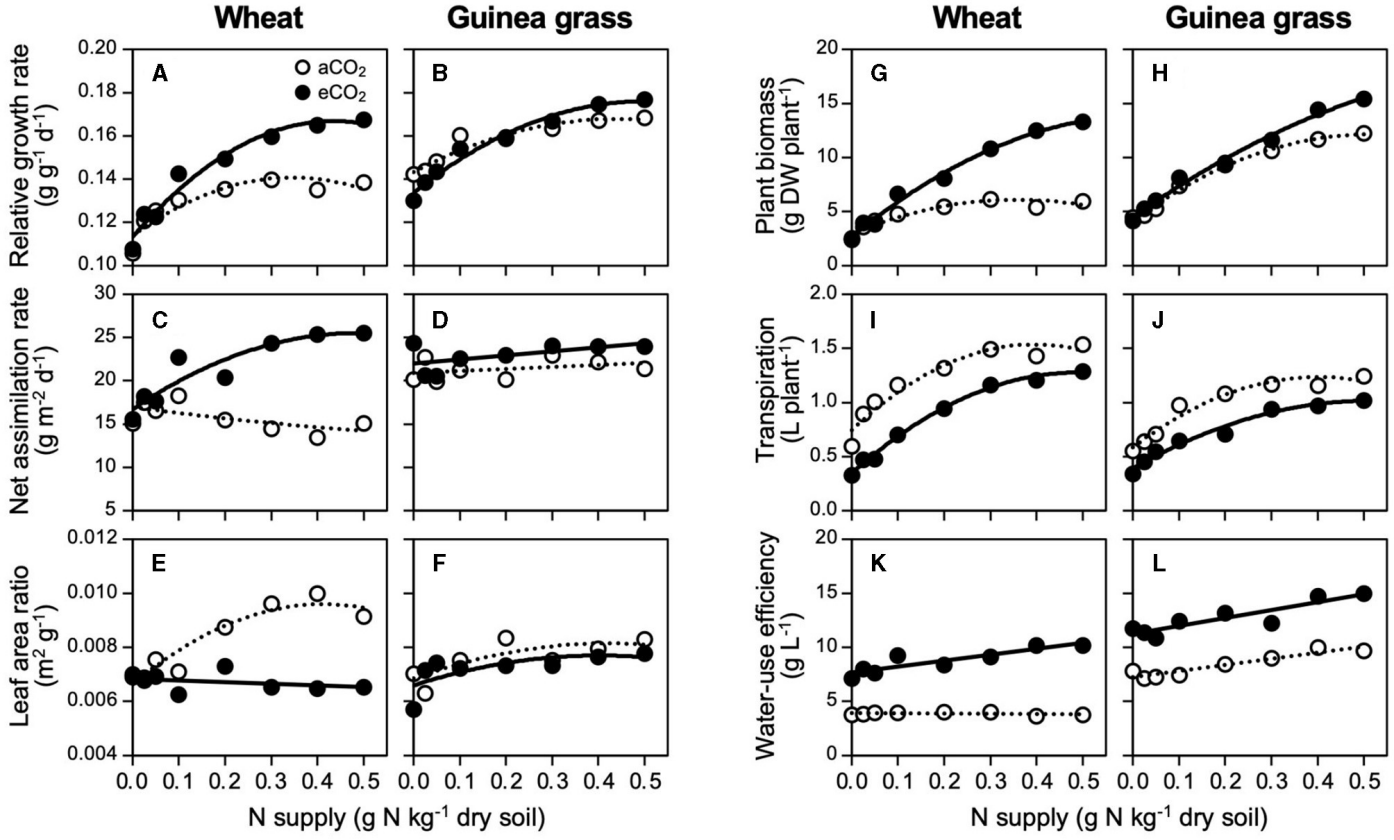
Growth responses for nitrate-N supply in C_3_ wheat and C_4_ guinea grass is grown for 28 d in the chambers under ambient (aCO_2_) or elevated (eCO_2_) CO_2_ treatments. Relative growth rate **(A,B)**, net assimilation rate **(C,D)**, leaf area ratio **(E,F)**, plant biomass **(G,H)**, cumulative transpiration **(I,J)**, and water-use efficiency **(K,L)**.

In response to the increase in N supply, both species increased their RGRs and attained maximum levels at 0.4 g N kg^−1^ soil for wheat in eCO_2_ and guinea grass in each CO_2_ treatment, but at a lower N supply level (0.3 g N kg^−1^ soil) in aCO_2_ wheat (Figures 2A,B). The eCO_2_ treatment significantly enhanced the RGR of wheat but not guinea grass (Table 3). In wheat, the enhancement of RGR by CO_2_ enrichment was attributable to a higher NAR (Figure 2C) rather than the LAR (Figure 2E), which supported the enhancement of foliar photosynthesis with increased N supply levels. At the highest NAR, wheat showed a higher RGR (i.e., 25 g m^−2^ d^−1^, Figure 2C) than that of guinea grass (Figure 2D). On the contrary, guinea grass showed less responses to CO_2_ enrichment and N supply in terms of RGR (Figure 2B), NAR (Figure 2D), and LAR (Figure 2F) than wheat.

**Table 3:** Regression equation and coefficient of determination (*R*^2^) of each parameter against nitrate-N supply in wheat and guinea grass grown for 28 d in the chambers under ambient (aCO_2_) or elevated (eCO_2_) CO_2_ treatments.

				**Relative growth rate (g g^**−1**^ day^**−1**^)**	**Net assimilation rate** **(g m^**−2**^ d^**−1**^)**	**Leaf area ratio (m^**2**^ g^**−1**^)**	**Plant biomass ** **(g DW plant^**−1**^)**	**Transpiration (L plant^**−1**^)**	**Water-use efficiency ** **(g L^**−1**^)**	**Leaf blade N conc.(g N m^**−2**^)**	**N acquisition per transpiration (mg N L^**−1**^)**	**Plant N content** **(mg N plant^**−1**^)**
Wheat	aCO2	Regression		y = −0.23x^2^ + 0.16x + 0.11	y = 3.3x^2^ – 7.1x + 17	y = −0.017x^2^ + 0.014x + 0.0066	y = −24x^2^ + 17x + 3.0	y = −5.1x^2^ + 4.0x + 0.74	y = −0.22x + 3.9	y = 3.3x + 0.65	y = 208x + 31	y = 369x + 24
		*R^2^*		0.847	0.409	0.899	0.903	0.934	0.104	0.983	0.983	0.992
	eCO2	Regression		y = −0.28x^2^ + 0.25x + 0.11	y = −37x^2^ + 36x + 18	y = −0.0006x + 0.0068	y = −27x^2^ + 35x + 2.7	y = −3.9x^2^ + 3.8x + 0.34	y = 5.5x + 7.7	y = 2.2x + 0.54	y = 257x + 55	y = 448.x + 16
		*R^2^*		0.964	0.86	0.118	0.989	0.994	0.802	0.989	0.975	0.992
		**ANOVA**	**CO**_**2**_	***P*** **=** **0.018**	***P*** **=** **0.013**	***P*** **=** **0.015**	***P*** **=** **0.029**	***P*** **<** **0.001**	***P*** **<** **0.001**	***P*** **=** **0.003**	***P*** **<** **0.001**	***P*** **=** **0.179**
			**N supply**	***P*** **=** **0.009**	***P*** **=** **0.765**	***P*** **=** **0.574**	***P*** **=** **0.084**	***P*** **<** **0.001**	***P*** **=** **0.571**	***P*** **<** **0.001**	***P*** **<** **0.001**	***P*** **<** **0.001**
Guinea grass	aCO2	Regression		y = −0.13x^2^ + 0.113x + 0.14	y = 2.3x + 21	y = −0.0072x^2^ + 0.0061x + 0.0069	y = −30x^2^ + 31x + 4.2	y = −4.0x^2^ + 3.2x + 0.59	y = 5.7x + 7.3	y = 1.2x + 0.88	y = 322x + 54	y = 473x + 32
		*R^2^*		0.915	0.13	0.609	0.993	0.953	0.883	0.842	0.987	0.997
	eCO2	Regression		y = −0.17x^2^ + 0.17x + 0.13	y = 4.7x + 22	y = −0.0069x^2^ + 0.0055x + 0.0066	y = −16x^2^ + 30x + 4.5	y = −2.4x^2^ + 2.4x + 0.39	y = 7.3x + 11	y = 1.3x + 0.83	y = 412x + 81	y = 529x + 29
		*R^2^*		0.975	0.322	0.494	0.989	0.975	0.816	0.867	0.985	0.999
		**ANOVA**	**CO**_**2**_	***P*** **=** **0.674**	***P*** **=** **0.049**	***P*** **=** **0.167**	***P*** **=** **0.037**	***P*** **<** **0.001**	***P*** **<** **0.001**	***P*** **=** **0.727**	***P*** **<** **0.001**	***P*** **=** **0.045**
			**N supply**	***P*** **=** **0.001**	***P*** **=** **0.400**	***P*** **=** **0.080**	***P*** **<** **0.001**	***P*** **<** **0.001**	***P*** **=** **0.002**	***P*** **=** **0.002**	***P*** **<** **0.001**	***P*** **<** **0.001**

*The bold values indicate probabilities by two-way analysis of variance (ANOVA)*.

The eCO_2_ treatment strongly enhanced the plant biomass under higher N levels, particularly in wheat, but had less of an effect on guinea grass (Figures 2G,H). As a result, CO_2_ enrichment resulted in a 2.2-fold increase in biomass in wheat but a small increase (i.e., by 1.3-fold) in guinea grass. Despite the increase in biomass in each species, water consumption during growth was always lower under the eCO_2_ treatment than under the aCO_2_ treatment across the N supply levels (Figures 2I,J). The lower water consumption but greater biomass was attributable to the enhanced WUE under the eCO_2_ treatment (Figures 2K,L) which ranged from 7 to 11 g biomass per liter of water in eCO_2_ wheat, remained constant at 4 g biomass per liter of water in aCO_2_ wheat, ranged from 11 to 15 g biomass per liter water in eCO_2_ guinea grass, and ranged from 7 to 10 g biomass per liter water in aCO_2_ guinea grass. Except for aCO_2_ wheat, the WUE increased under high N supply levels (Table 3).

**Figure 3:**
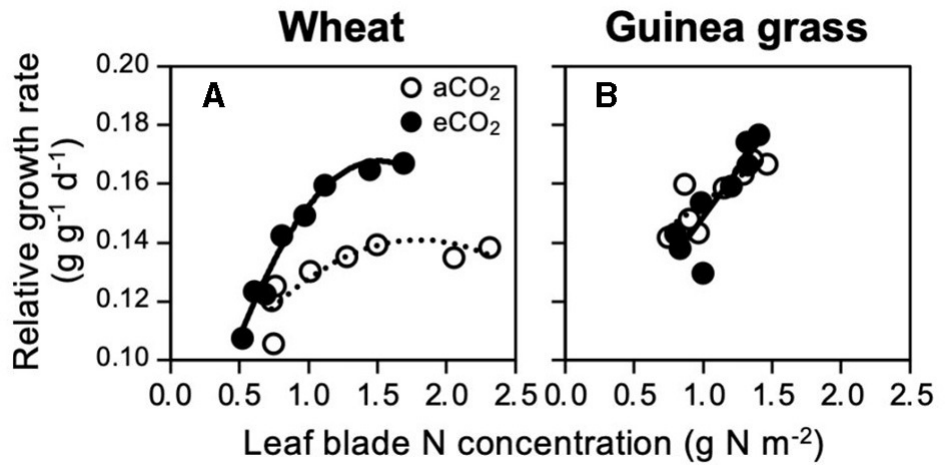
Relationships between relative growth rate and foliar N concentration in C_3_ wheat **(A)** and C_4_ guinea grass **(B)** grown for 28 d in the chambers under ambient (aCO_2_) or elevated (eCO_2_) CO_2_ treatments.

To assess if the foliar N demand for maximum growth is affected by the CO_2_ treatments, RGR was regressed against the foliar N concentration (Figure 3). To represent the foliar N concentration, we used the area basis unit (mg N m^−2^ leaf area) instead of the mass basis unit (mg N g^−1^ leaf dry matter) because the latter would not be suitable, especially when the leaf mass per area is affected by the CO_2_ treatment (Yi et al., 2020), as observed in the present study (Supplementary Figure 2). Under both CO_2_ treatments, wheat showed saturated RGRs against the foliar N concentration (Figure 3A), but guinea grass did not (Figure 3B). Both species showed maximum RGRs approximately at 1.5 g N m^−2^ irrespective of the CO_2_ treatments, which indicated that eCO_2_ would not increase the N demand for the maximum growth.

**Figure 4:**
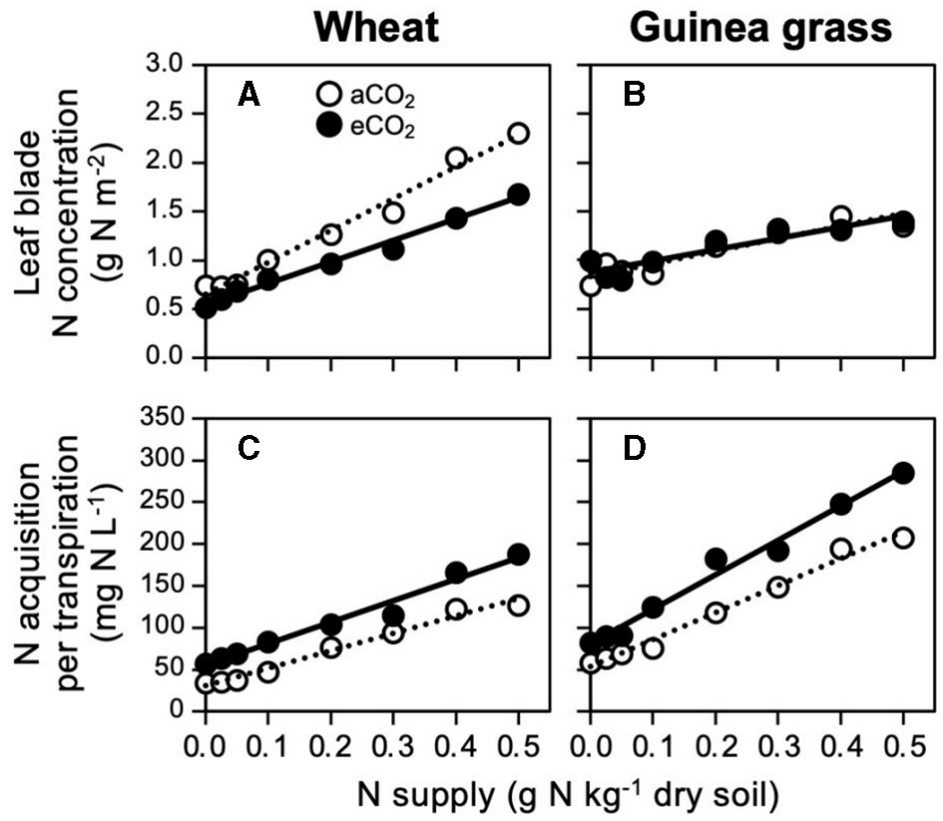
Relationships between foliar N concentration **(A,B)** or N acquisition per transpiration **(C,D)** and nitrate-N supply in C_3_ wheat and C_4_ guinea grass grown for 28 d in the chambers under ambient (aCO_2_) or elevated (eCO_2_) CO_2_ treatments.

However, to reach a foliar N concentration of 1.5 g N m^−2^, the level of N supply to the soil differed between the CO_2_ treatments in wheat (Figure 4A) because the slope of the N concentration against the N supply was 1.5-fold steeper in aCO_2_ wheat than in eCO_2_ wheat (Table 3). As a result, in wheat, 0.4 g N kg^−1^ soil was required to reach the foliar N concentration of 1.5 g N m^−2^ under eCO_2_ treatment although a lower N supply (0.3 g N kg^−1^ soil) was sufficient under aCO_2_ treatment. In guinea grass, such a difference in the slope was very small in response to the CO_2_ treatments (Table 3), resulting in similar N supply requirements to reach certain foliar N concentrations (Figure 4B). The lower slope in eCO_2_ wheat did not result from decreased N acquisition with decreased transpiration (Figure 2I) because the eCO_2_ treatment enabled higher N acquisition levels per water consumption than the aCO_2_ treatment across all N supply levels (Figure 4C). Additionally, guinea grass also showed higher N acquisition per unit of transpired water at any N supply level at eCO_2_ (Figure 4D), but the slope of the regression line was steeper than wheat (Table 3). Consequently, we observed that eCO_2_ increased N acquisition per unit of transpired water, which did not depend only on the species (Table 1) but also on the N supply level (Figures 4C,D).

**Figure 5:**
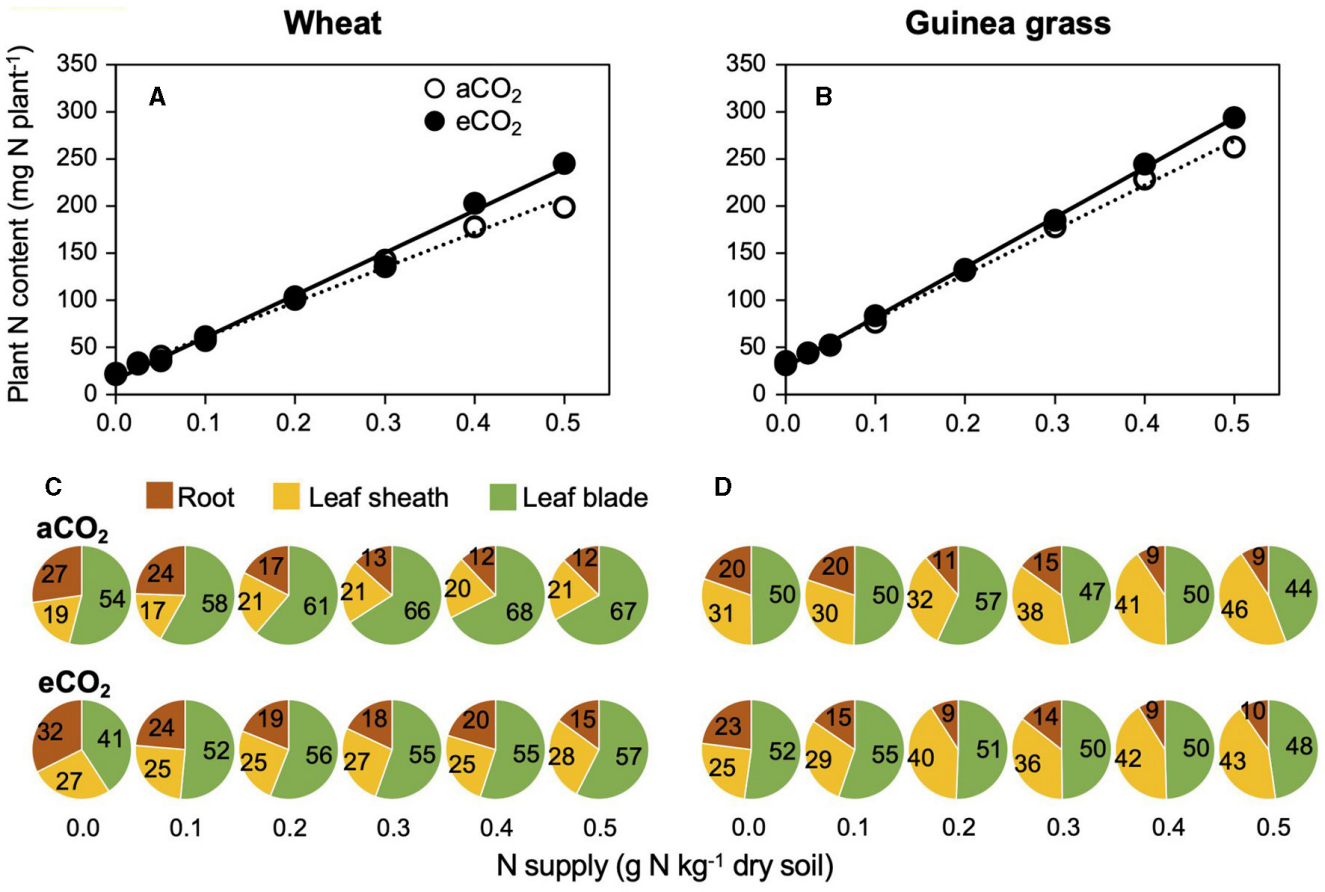
Relationships between plant N accumulation and nitrate-N supply **(A,B)**, N distribution to each organ **(C,D)** in C_3_ wheat and C_4_ guinea grass grown for 28 d in the chambers under ambient (aCO_2_) or elevated (eCO_2_) CO_2_ treatments. Each value in the pie chart **(C,D)** shows the percentage within the individual plant.

The total N content, including small N accumulation before transplanting in addition to large amounts of N, acquired during 28-day growth, was similar at lower N supply rates or higher at higher N supply rates under eCO_2_ treatments compared to aCO_2_ treatments in both species (Figures 5A,B). However, the distribution pattern of N to each organ (i.e., leaf blade, leaf sheath, and root) differed remarkably due to the CO_2_ and N treatments and was dependent on the species (Figures 5C,D). An increased N supply decreased the N distribution to the roots in both species, although wheat plants had relatively higher N contents in their roots than guinea grass, especially at eCO_2_. In response to CO_2_ enrichment, wheat increased the N distribution to the leaf sheath and decreased the N distribution to the leaf blade. In guinea grass, however, CO_2_ enrichment did not affect the N distribution in the leaves.

## Discussion

### Does eCO_2_ Promote Nitrate Accumulation in Plants?

Currently, there are different views on whether eCO_2_ inhibits nitrate assimilation in C_3_ plants (Bloom et al., 2020) or not (Andrews et al., 2020). Inhibition of nitrate assimilation under eCO_2_ results in nitrate accumulation. Hence, less organic-N could be present in plants when total N content was similar. Indeed, our results showed that eCO_2_ significantly decreased organic-N concentrations (Supplementary Table 4), except in *Amaranthus*, without an increase in biomass (Figures 1A,B). However, it is difficult to distinguish whether the apparent decrease in organic-N concentration (organic-N content per biomass) under eCO_2_ means a shortage of organic-N or a consequence of dilution due to biomass increase. To eliminate the effect of biomass increase, we used the percentage of nitrate-N in total N (nitrate-N content per total N content) as an index of nitrate accumulation. As a result, in most species, we found that eCO_2_ decreased (i.e., wheat, potato, and guinea grass) or did not change (i.e., *Amaranthus*) nitrate accumulation at the whole-plant level under the nitrate-fed condition (Figure 1E; Table 2), which likely supports the view of Andrews et al. (2019, 2020).

Organic-N in the shoot is derived from not only the assimilation of shoot nitrate but also the import of amino acids generated by nitrate assimilation in the root (Andrews, 1986). Thus, shoot nitrate reductase activities and shoot organic-N concentrations alone may not be accurate estimates of shoot nitrate assimilation (Bloom et al., 2020). It has also been proposed that eCO_2_ decreased nitrate assimilation in the shoot but enhanced it in the root (Bloom et al., 2020), which emphasizes the importance to distinguish between the shoot and the root. However, considering the percentage of nitrate-N in each organ (Supplementary Table 5), we could not confirm the enhancement of nitrate accumulation in the nitrate-fed plants under eCO_2_ in most species, except in rice.

Only rice showed a significant but slight increase in plant nitrate accumulation in response to CO_2_ enrichment (Figure 1E; Table 2), along with decreased δ^15^N values in the residues after nitrate extraction (Supplementary Table 5), suggesting that eCO_2_ inhibited nitrate reductase activities. While the results support the views of Bloom et al. (2020), an important issue is whether such nitrate accumulation could inhibit growth. We expected that if plants in the soil could receive not only ${\text{NO}}_{3}^{-}$ but also reduced-N, such as urea and ${\text{NH}}_{4}^{+}$, they would be less dependent on nitrate assimilation, and consequently, the growth would improve, especially in rice, because it prefers ammonium nutrition. As expected, the percentage of nitrate-N in total N decreased in the urea-fed plants compared with that in the nitrate-fed plants (Figures 1E,F; Table 2), especially in the C_3_ species, including rice. Nevertheless, there was no significant improvement in the biomass (Figure 1B; Table 2) and RGR (Table 1) due to reduced nitrate accumulation in rice. Therefore, such a slight increase in nitrate accumulation would not cause growth inhibition compared with that in the urea-fed rice (Table 1). As a result, it was difficult to confirm that eCO_2_ limits plant growth *via* inhibition of nitrate assimilation in any of the five plant species used, at least under the conditions of this study.

### Does eCO_2_ Lower Nitrate Acquisition by Plants?

It has been hypothesized that transpiration reduced by eCO_2_ may reduce nitrate-N acquisition (Taub and Wang, 2008; Feng et al., 2015; Tausz-Posch et al., 2020). This hypothesis seemed to involve an assumption that the amount of N acquired per transpired water is not affected by eCO_2_. Otherwise, it would be difficult to predict a decrease in N acquisition only from the decrease in transpiration. However, we found that the amount of N acquired per transpired water increased under eCO_2_, regardless of plant species (Table 1) and the level of N supply in wheat and guinea grass (Figures 4C,D). The results revealed that the above assumption may not be suitable. Considering that the enhancement of nitrate-N acquisition per transpiration at eCO_2_ was consistent among the species at *p* = 0.013 (Table 1), the changes in leaf area, root length, or the ratio by CO_2_ enrichment (Supplementary Table 1), which were not consistent among the species, would not explain the enhancement.

Furthermore, the enhancement of nitrate-N acquisition per transpiration under eCO_2_ was consistently observed even with no increase in the biomass not only in *Amaranthus* in Experiment 1 (Table 1) but also in wheat or guinea grass at lower N supply rates in Experiment 2 (Figures 4C,D). The results confirmed that enhancement was independent of the growth promotion. In fact, a parallel slope of the regression, but elevated intercept at eCO_2_, in each species (Figures 4C,D) suggests that eCO_2_ can increase the conductance of nitrate-N from soil to plant to a constant level, independent of the level of N supply that strongly affected the RGR of each species (Figures 4A,B).

In a meta-analysis using data from several free-air CO_2_ enrichment (FACE) experiments, Feng et al. (2015) focused on the fact that N concentrations decreased with eCO_2_ even when biomass did not increase (i.e., lower N content). The phenomenon was one of the reasons that they claimed a reduction in nitrate-N acquisition per transpiration under eCO_2_. However, in our results (lower levels of nitrate-N supply in Experiment 2), when plant biomass was comparable between eCO_2_ and aCO_2_ treatments (Figures 2G,H), N content was also comparable (Figures 5A,B). One possible reason for the discrepancy is that our measurements comprised “whole plants” at the individual level, and their results were mainly derived from “aboveground parts” at the ecosystem level. For example, in Experiment 2, wheat without N supply allocated 73% of total N to the shoots under aCO_2_, but only 68% of that under eCO_2_ (Figure 5C), even though the N content of the whole plant was similar (Figure 5A). Perhaps, a problem with FACE experiments may be the difficulty to measure the belowground parts accurately.

To the best of our knowledge, this is the first study to clarify that eCO_2_ likely enhances the nitrate-N acquisition per unit transpiration consistently across the species and N supply levels by measuring cumulative transpiration precisely at the individual plant level, although a similar phenomenon was also observed by Houshmandfar et al. (2018) in wheat at a field level. Consequently, we could not confirm that nitrate-N acquisition decreases under eCO_2_, despite the lower transpiration observed in various species. Therefore, our results indicated that the N concentration decreased under eCO_2_ (Figures 1C,D), but the reason could not be explained by a lower N acquisition even if transpiration was lowered by CO_2_ enrichment.

### Does the Dilution Effect Explain the Decrease in N Concentration in Plants?

The commonly observed decline in plant N concentration under eCO_2_ treatments has frequently been interpreted as a dilution effect (Poorter et al., 1997; Gifford et al., 2000; Taub and Wang, 2008; Tausz-Posch et al., 2020), which results from a higher carbon assimilation rate than N acquisition rate (i.e., growth dilution). Consequently, plant tissue N concentrations usually decrease under eCO_2_ at both the foliar and whole-plant levels (Ainsworth and Long, 2005). In Experiment 1, our results clearly showed that eCO_2_ decreased the plant N concentration irrespective of the form of N-fertilizer, except in C_4_ *Amaranthus* (Figures 1C,D; Table 2). According to Taub and Wang (2008), biomass dilution occurs whenever there is a higher increase in the total biomass of a plant under eCO_2_ treatments relative to growth under aCO_2_ treatments than the corresponding increase in the total N. This agreed with the species investigated in the present study, including *Amaranthus*, which showed no significant effects of eCO_2_ on the plant N concentration, the biomass (Figures 1A,B; Table 2), and growth rate (Table 1). Therefore, our results fully support that the dilution effect causes a decrease in the N concentration.

However, it remained unclear if such a decrease in the N concentration limits plant growth under eCO_2_ treatments. In fact, the foliar N concentration on the area basis (Supplementary Figures 1A,B) revealed no effect by CO_2_ enrichment. Therefore, we can consider that an apparent decrease in the mass-based N concentration was merely the result of the increase in leaf mass per area due to eCO_2_ (Supplementary Figures 1C,D). To address this, we further investigated the growth responses of wheat and guinea grass (as C_3_ and C_4_ representatives, respectively) to nitrate-N supply under both CO_2_ treatments in Experiment 2.

### What Is the Quantity of N Supply That Is Required for the Full Growth at eCO_2_?

While the problem of N-limitation under eCO_2_ has been highlighted (Poorter et al., 1997; Cotrufo et al., 1998; Gifford et al., 2000; Taub and Wang, 2008; Feng et al., 2015), the actual N requirement for the maximum growth under eCO_2_ has rarely been quantified as Conroy (1992) and Yi et al. (2020). While eCO_2_ significantly increased the RGR in wheat but not in guinea grass (Table 3), each species showed an increase in the RGR with a higher N supply, and then peaked when there was no longer N-limitation (Figures 2A,B). To achieve the maximum RGR, wheat required a 1.3-fold N supply under eCO_2_ compared with that under aCO_2_ (Figure 2A) accompanying a 2.2-fold biomass increase (Figure 2G). However, the N requirement by guinea grass was less affected by the CO_2_ treatment (Figure 2B). The results revealed that accelerated RGR by eCO_2_ could create a demand for more N in wheat, causing the N-limited growth unless additional N was supplied.

CO_2_ enrichment changed the wheat growth from LAR-dependent to NAR-dependent, in which the leaf N concentration strongly determined the RGR (Figure 3A), but this did not occur in guinea grass (Figures 2D,F). Similar results were reported by Imai and Murata (1979) using C_3_ plants (rice and soybean) and C_4_ plants (maize and Japanese millet). According to the meta-analysis by Poorter and Navas (2003), eCO_2_ increased NAR (+24% on average) but decreased LAR (−13% on average) across the species in vegetable growth, which seems to be consistent with eCO_2_ wheat (Figures 2C,E). In contrast, the growth of aCO_2_ wheat was LAR-dependent (Figure 2E), which is a typical trait for fast-growing species at the current CO_2_ level (Poorter and Navas, 2003). Despite the LAR-dependent growth in aCO_2_ wheat, N supply levels above 0.3 g N kg^−1^ soil could no longer increase the leaf area with the saturated tiller number (Supplementary Figure 3), thus, exhibiting growth limitation by CO_2_ rather than N as eCO_2_ further increased the number of tillers and leaf area.

Burnett et al. (2018) compared fast-growing domesticated annual barley with a slow-growing wild perennial relative under different levels of nutrient supply. They found that the perennial barley has a higher amino acid/sucrose ratio than the annual, implying a greater carbon source-limitation in the perennial than the annual barley. Indeed, eCO_2_ alleviating the source-limitation weakly increased photosynthesis in the annual but strongly increased photosynthesis and sink (tiller) development in the perennial, again suggesting that the growth was sink-limited in the annual but source-limited in the perennial (Burnett et al., 2016). Our results suggest that more N supply than the sufficient level under aCO_2_ along with eCO_2_ may alleviate the sink-limitation in wheat (Supplementary Figure 3).

It was notable that the eCO_2_ treatment resulted in more than a two-fold increase in wheat biomass despite the lower water consumption compared to aCO_2_ wheat (Figure 2I) with strongly elevated WUE (Figure 2K), which was comparable to guinea grass (Figure 2L). Such enhanced WUE, accompanied by a higher dry matter, was also observed in other species used under eCO_2_ in Experiment 1 (Table 1), which hardly occurs at the current CO_2_ because of the tight coupling between transpiration and carbon assimilation, except during an improvement to reduce nocturnal transpiration (Coupel-Ledru et al., 2016). The growth performances of wheat under eCO_2_ were equivalent to those of C_4_ guinea grass (Figures 2B,H,J,L), which revealed that eCO_2_ may enable C_4_ performances by C_3_ wheat without genetic alteration. Consequently, the eCO_2_ levels are likely to make C_3_ plants less dependent on water to acquire both CO_2_ and N but more dependent on the N supply, regardless of the N form of the fertilizer.

### Where Does the Increase in N Demand Occur?

Although the RGR showed saturated responses against foliar N concentration in wheat (Figure 3A), the minimum N concentration for the maximum RGR was lower or similar under eCO_2_ than under aCO_2_ (Figure 3A), suggesting that the N demand at the foliar level for the maximum RGR was not necessarily increased by CO_2_ enrichment. Indeed, the distribution of N to the leaf blade was always lower under eCO_2_ than under aCO_2_ in wheat (Figure 5C), despite the similar whole-plant N contents between eCO_2_ and aCO_2_ (Figure 5A). The results suggest decreased foliar N demand in C_3_ wheat under eCO_2_ as a result of decreased investment in photosynthetic and photorespiratory enzymes (Davey et al., 1999; Stitt and Krapp, 1999; Gifford et al., 2000; Long et al., 2004; McMurtrie et al., 2008).

Regardless of the reduced N demand at the foliar level in wheat, eCO_2_ did not necessarily decrease the N requirement for the maximum RGR at the whole-plant level (Figure 2A). This could be attributed to the lower response of the foliar N concentration to N supply at eCO_2_ than at aCO_2_ (Figure 4A), which did not occur in guinea grass (Figure 4B). This interspecific difference may be explained by an increased N requirement by other organs, particularly the leaf sheath under eCO_2_ (Figure 5C), which was not observed in guinea grass having an inherently greater N distribution in leaf sheath (Figure 5D). To explain these findings, it was assumed that the role of the leaf sheath to store and temporally accumulate carbohydrates would be more important for wheat under eCO_2_, and thus, the export of carbohydrates accumulated in leaf blade would be accelerated to alleviate the downregulation of photosynthesis (Stitt, 1991; Ainsworth and Bush, 2011).

## Conclusions

We showed that inhibited nitrate assimilation, which was weakly observed only in rice, cannot explain the growth limitation by N induced under eCO_2_ in any species, including rice. Furthermore, we found that nitrate acquisition is not necessarily reduced, despite a decrease in transpiration under eCO_2_, because of an increase in nitrate acquisition per unit water transpired. Consequently, it is likely difficult to alleviate the N-limitation by feeding with urea instead of nitrate. Our results for all species did not contradict the dilution-effect hypothesis, suggesting that a higher N supply is essential to overcome the N-limitation. Thus, we assessed the minimum nitrate-N supply for the maximum growth of wheat and found that eCO_2_ resulted in a 2.2-fold increase in wheat biomass with a 1.3-fold N supply compared to aCO_2_. Surprisingly, this greater biomass was achieved with lower water consumption. We, therefore, concluded that eCO_2_ strengthens the N-limitation with an accelerated plant growth rate but may enable an increase in biomass with a lower water consumption by meeting the N demand, regardless of the fed-form.

It should be noted that our results were obtained under steady day-light (400 μmol m^−2^ s^−1^), which is not sufficiently high to saturate photosynthesis, especially under the eCO_2_ treatment and for the C_4_ species examined in this study. Considering that plant responses to eCO_2_ depend on irradiance levels (Wheeler et al., 1991; Ghannoum et al., 1997; Paterson et al., 1999), it would be worthwhile to test whether our conclusions are valid under different light intensities.

## Data Availability Statement

The original contributions presented in the study are included in the article/Supplementary Material, further inquiries can be directed to the corresponding author.

## Author Contributions

KY designed research. MI performed research with contributions from YY. MI, YY, and KY analyzed data. MI and KY wrote the paper. YY revised it. All authors contributed to the article and approved the submitted version.

## Funding

This study was supported by Grant for Environmental Research Projects from The Sumitomo Foundation, and in part by JSPS KAKENHI Grant Number 21H02328.

## Conflict of Interest

The authors declare that the research was conducted in the absence of any commercial or financial relationships that could be construed as a potential conflict of interest.

## Publisher's Note

All claims expressed in this article are solely those of the authors and do not necessarily represent those of their affiliated organizations, or those of the publisher, the editors and the reviewers. Any product that may be evaluated in this article, or claim that may be made by its manufacturer, is not guaranteed or endorsed by the publisher.
