# Supplementary material for: Revisiting Why Plants Become N Deficient Under Elevated CO2: Importance to Meet N Demand Regardless of the Fed-Form
Source: Front Plant Sci. 2021 Nov 4;12:726186. doi: 10.3389/fpls.2021.726186 (PMC8600045; doi:10.3389/fpls.2021.726186)
Supplement: Supplementary file 1 [file Data_Sheet_1.zip › Supplementary Figures and Tables.pdf]

Table S1. Leaf area, root length and leaf area per root length in nitrate-fed or urea-fed plants in five species grown for 28 d in the chambers under ambient (aCO_2) or elevated (eCO_2) CO_2 treatments.

Species	CO_2	N-fed form	Leaf area (cm^2 $plant^{-1}$)	Root length (m $plant^{-1}$)	Leaf area / root length ratio (cm^2 m^{-1})
Wheat	aCO_2	Nitrate	874 ± 15	222 ± 15	4.01 ± 0.25
		Urea	806 ± 36	208 ± 26	4.06 ± 0.41
	eCO_2	Nitrate	832 ± 23	315 ± 48	3.00 ± 0.58
		Urea	945 ± 47	238 ± 30	4.22 ± 0.50
	**ANOVA**	**CO_2 (C)**	***P* = 0.167**	***P* = 0.124**	***P* = 0.432**
		**N form (N)**	***P* = 0.497**	***P* = 0.242**	***P* = 0.244**
		**C X N**	***P* = 0.017**	***P* = 0.410**	***P* = 0.287**
Rice	aCO_2	Nitrate	148 ± 9	98 ± 6	1.52 ± 0.03
		Urea	145 ± 15	86 ± 10	1.72 ± 0.06
	eCO_2	Nitrate	172 ± 18	138 ± 7	1.27 ± 0.12
		Urea	213 ± 19	144 ± 19	1.52 ± 0.07
	**ANOVA**	**CO_2 (C)**	***P* = 0.013**	***P* = 0.003**	***P* = 0.030**
		**N form (N)**	***P* = 0.250**	***P* = 0.837**	***P* = 0.029**
		**C X N**	***P* = 0.195**	***P* = 0.516**	***P* = 0.781**
Potato	aCO_2	Nitrate	561 ± 22	148 ± 7	3.82 ± 0.18
		Urea	556 ± 17	149 ± 6	3.75 ± 0.21
	eCO_2	Nitrate	537 ± 18	155 ± 9	3.49 ± 0.18
		Urea	559 ± 26	153 ± 4	3.68 ± 0.21
	**ANOVA**	**CO_2 (C)**	***P* = 0.615**	***P* = 0.477**	***P* = 0.389**
		**N form (N)**	***P* = 0.702**	***P* = 0.943**	***P* = 0.802**
		**C X N**	***P* = 0.520**	***P* = 0.775**	***P* = 0.571**
Guinea grass	aCO_2	Nitrate	961 ± 44	264 ± 22	3.76 ± 0.39
		Urea	974 ± 29	225 ± 15	4.40 ± 0.32
	eCO_2	Nitrate	1095 ± 27	254 ± 27	4.47 ± 0.41
		Urea	1114 ± 78	217 ± 27	5.66 ± 1.10
	**ANOVA**	**CO_2 (C)**	***P* = 0.017**	***P* = 0.743**	***P* = 0.205**
		**N form (N)**	***P* = 0.750**	***P* = 0.185**	***P* = 0.238**
		**C X N**	***P* = 0.953**	***P* = 0.972**	***P* = 0.719**
Amaranthus	aCO_2	Nitrate	918 ± 13	276 ± 19	3.39 ± 0.21
		Urea	937 ± 45	215 ± 26	4.62 ± 0.56
	eCO_2	Nitrate	827 ± 80	254 ± 52	4.14 ± 1.10
		Urea	908 ± 30	200 ± 21	4.72 ± 0.45
	**ANOVA**	**CO_2 (C)**	***P* = 0.244**	***P* = 0.636**	***P* = 0.589**
		**N form (N)**	***P* = 0.325**	***P* = 0.155**	***P* = 0.264**
		**C X N**	***P* = 0.540**	***P* = 0.923**	***P* = 0.680**

Each data is mean ± SE (n = 4).

Table S2. Biomass in each organ in nitrate-fed or urea-fed plants in five species grown for 28 d in the chambers under ambient (aCO_2) or elevated (eCO_2) CO_2 treatments.

Species	CO_2	N-fed form	Biomass (g DW plant^{-1})				
			Leaf	Sheath or stem	Shoot	Tuber	Root
Wheat	aCO_2	Nitrate	3.11 ± 0.12	1.22 ± 0.09	4.34 ± 0.20		2.69 ± 0.38
		Urea	3.17 ± 0.19	1.36 ± 0.08	4.53 ± 0.27		3.23 ± 1.14
	eCO_2	Nitrate	4.14 ± 0.27	2.00 ± 0.14	6.14 ± 0.40		4.51 ± 1.35
		Urea	4.06 ± 0.13	2.29 ± 0.46	6.35 ± 0.44		3.86 ± 0.92
	**ANOVA**	**CO_2 (C)**	**P < 0.001**	**P = 0.005**	**P < 0.001**		**P = 0.252**
		**N form (N)**	**P = 0.968**	**P = 0.408**	**P = 0.558**		**P = 0.956**
		**C × N**	**P = 0.725**	**P = 0.760**	**P = 0.975**		**P = 0.566**
Rice	aCO_2	Nitrate	0.73 ± 0.05	0.77 ± 0.07	1.50 ± 0.11		0.42 ± 0.04
		Urea	0.75 ± 0.08	0.74 ± 0.08	1.49 ± 0.16		0.35 ± 0.05
	eCO_2	Nitrate	1.04 ± 0.08	1.18 ± 0.10	2.22 ± 0.18		0.62 ± 0.06
		Urea	1.26 ± 0.11	1.50 ± 0.17	2.76 ± 0.27		0.71 ± 0.11
	**ANOVA**	**CO_2 (C)**	**P < 0.001**	**P < 0.001**	**P < 0.001**		**P = 0.002**
		**N form (N)**	**P = 0.162**	**P = 0.234**	**P = 0.190**		**P = 0.912**
		**C × N**	**P = 0.230**	**P = 0.155**	**P = 0.172**		**P = 0.272**
Potato	aCO_2	Nitrate	1.88 ± 0.09	0.89 ± 0.04	2.77 ± 0.12	1.87 ± 0.23	0.46 ±0.02
		Urea	2.03 ± 0.03	0.89 ± 0.03	2.92 ± 0.05	2.60 ± 0.16	0.53 ± 0.03
	eCO_2	Nitrate	2.08 ± 0.13	0.91 ± 0.05	2.99 ± 0.16	3.28 ± 0.47	0.51 ±0.03
		Urea	2.13 ± 0.05	0.83 ± 0.06	2.96 ± 0.02	3.77 ± 0.48	0.49 ±0.03
	**ANOVA**	**CO_2 (C)**	**P = 0.092**	**P = 0.672**	**P = 0.235**	**P = 0.004**	**P = 0.963**
		**N form (N)**	**P = 0.253**	**P = 0.421**	**P = 0.576**	**P = 0.117**	**P = 0.349**
		**C × N**	**P = 0.519**	**P = 0.475**	**P = 0.410**	**P = 0.745**	**P = 0.090**
Guinea grass	aCO_2	Nitrate	4.30 ± 0.19	4.59 ± 0.20	8.89 ± 0.39		2.51 ± 0.31
		Urea	4.39 ± 0.14	4.74 ± 0.15	9.13 ± 0.19		2.10 ± 0.12
	eCO_2	Nitrate	5.52 ± 0.26	5.47 ± 0.16	10.99 ± 0.41		2.54 ± 0.15
		Urea	5.34 ± 0.38	5.35 ± 0.22	10.69 ± 0.43		2.13 ± 0.30
	**ANOVA**	**CO_2 (C)**	**P = 0.001**	**P = 0.002**	**P < 0.001**		**P = 0.884**
		**N form (N)**	**P = 0.871**	**P = 0.936**	**P = 0.941**		**P = 0.108**
		**C × N**	**P = 0.607**	**P = 0.478**	**P = 0.474**		**P = 1.000**
Amaranthus	aCO_2	Nitrate	4.13 ± 0.15	2.22 ± 0.11	6.35 ± 0.23		1.16 ± 0.08
		Urea	3.52 ± 0.24	1.54 ± 0.14	5.06 ± 0.36		0.92 ± 0.16
	eCO_2	Nitrate	3.91 ± 0.49	2.28 ± 0.28	6.19 ± 0.69		1.21 ± 0.21
		Urea	3.49 ± 0.06	1.69 ± 0.12	5.17 ± 0.14		0.96 ± 0.04
	**ANOVA**	**CO_2 (C)**	**P = 0.671**	**P = 0.566**	**P = 0.964**		**P = 0.753**
		**N form (N)**	**P = 0.093**	**P = 0.004**	**P = 0.016**		**P = 0.099**
		**C × N**	**P = 0.746**	**P = 0.825**	**P = 0.751**		**P = 0.985**

Each data is mean ± SE (n = 4).

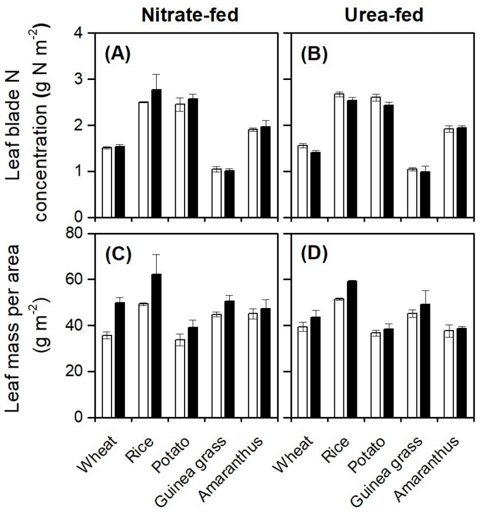

Figure S1. Foliar N concentration (A, B) and leaf mass per area (C, D) length in nitrate-fed or urea-fed plants in five species grown for 28 d in the chambers under ambient (aCO_2) or elevated (eCO_2) CO_2. treatments

Table S3. Total N concentration in each organ in nitrate-fed or urea-fed plants in five species grown for 28 d in the chambers under ambient (aCO_2) or elevated (eCO_2) CO_2 treatments.

Species	CO_2	N-fed form	Nitrogen concentration (mg N g^{-1} DW)				
			Leaf	Sheath or stem	Shoot	Tuber	Root
Wheat	aCO_2	Nitrate	42.62 ± 1.79	30.41 ± 3.07	39.20 ± 2.17		13.79 ± 0.84
		Urea	39.75 ± 1.68	25.46 ± 2.12	35.46 ± 1.76		13.61 ± 1.96
	eCO_2	Nitrate	31.16 ± 2.33	18.78 ± 2.17	27.14 ± 2.29		11.55 ± 1.41
		Urea	32.72 ± 1.41	19.78 ± 2.10	28.00 ± 1.83		11.90 ± 1.53
	**ANOVA**	**CO_2 (C)**	***P* < 0.001**	***P* < 0.001**	***P* < 0.001**		***P* = 0.209**
		**N form (N)**	***P* = 0.725**	***P* = 0.427**	***P* = 0.491**		***P* = 0.955**
		**C × N**	***P* = 0.251**	***P* = 0.240**	***P* = 0.279**		***P* = 0.862**
Rice	aCO_2	Nitrate	50.70 ± 0.66	26.77 ± 0.67	38.41 ± 0.75		15.33 ± 0.70
		Urea	52.04 ± 0.95	27.78 ± 0.46	39.95 ± 0.71		17.74 ± 1.01
	eCO_2	Nitrate	44.76 ± 0.84	24.18 ± 0.83	33.86 ± 0.51		14.97 ± 0.19
		Urea	42.93 ± 1.42	23.65 ± 0.97	32.55 ± 1.44		16.21 ± 0.05
	**ANOVA**	**CO_2 (C)**	***P* < 0.001**	***P* = 0.001**	***P* < 0.001**		***P* = 0.153**
		**N form (N)**	***P* = 0.812**	***P* = 0.755**	***P* = 0.902**		***P* = 0.012**
		**C × N**	***P* = 0.142**	***P* = 0.331**	***P* = 0.149**		***P* = 0.361**
Potato	aCO_2	Nitrate	73.18 ± 1.72	51.28 ± 1.88	66.06 ± 1.41	21.12 ± 1.81	21.83 ± 1.09
		Urea	71.06 ± 0.51	38.04 ± 1.93	61.03 ± 0.86	18.47 ± 0.87	22.11 ± 1.29
	eCO_2	Nitrate	66.34 ± 3.29	41.23 ± 3.80	58.72 ± 3.27	18.22 ± 1.53	22.22 ± 1.20
		Urea	63.97 ± 3.30	33.79 ± 2.80	55.46 ± 2.63	15.88 ± 0.75	21.19 ± 0.17
	**ANOVA**	**CO_2 (C)**	***P* = 0.016**	***P* = 0.022**	***P* = 0.014**	***P* = 0.059**	***P* = 0.801**
		**N form (N)**	***P* = 0.386**	***P* = 0.003**	***P* = 0.091**	***P* = 0.082**	***P* = 0.726**
		**C × N**	***P* = 0.962**	***P* = 0.306**	***P* = 0.701**	***P* = 0.908**	***P* = 0.538**
Guinea grass	aCO_2	Nitrate	23.40 ± 1.10	15.85 ± 0.88	19.50 ± 0.97		13.01 ± 0.64
		Urea	23.23 ± 0.55	17.03 ± 0.50	20.01 ± 0.22		13.77 ± 1.20
	eCO_2	Nitrate	20.02 ± 0.77	13.55 ± 0.55	16.80 ± 0.67		13.77 ± 0.97
		Urea	20.02 ± 0.62	13.46 ±0.72	16.76 ± 0.51		14.26 ± 1.18
	**ANOVA**	**CO_2 (C)**	***P* = 0.001**	***P* = 0.001**	***P* = 0.001**		***P* = 0.551**
		**N form (N)**	***P* = 0.914**	***P* = 0.440**	***P* = 0.724**		***P* = 0.554**
		**C × N**	***P* = 0.916**	***P* = 0.368**	***P* = 0.688**		***P* = 0.901**
Amaranthus	aCO_2	Nitrate	42.57 ± 1.90	22.96 ± 1.36	35.74 ± 1.63		19.46 ± 0.56
		Urea	51.61 ± 3.77	32.84 ± 3.89	45.93 ± 3.95		21.10 ± 1.47
	eCO_2	Nitrate	42.09 ± 2.78	27.61 ± 5.64	36.86 ± 3.73		20.41 ± 1.76
		Urea	50.55 ± 1.06	29.68 ± 0.48	43.79 ± 0.84		22.17 ± 0.76
	**ANOVA**	**CO_2 (C)**	***P* = 0.771**	***P* = 0.836**	***P* = 0.861**		***P* = 0.430**
		**N form (N)**	***P* = 0.005**	***P* = 0.114**	***P* = 0.011**		***P* = 0.194**
		**C × N**	***P* = 0.914**	***P* = 0.287**	***P* = 0.580**		***P* = 0.964**

Each data is mean ± SE (n = 4).

Table S4. Organic-N concentration in each organ in nitrate-fed or urea-fed plants in five species grown for 28 d in the chambers under ambient (aCO_2) or elevated (eCO_2) CO_2 treatments.

Species	CO_2	N-fed form	Organic nitrogen concentration (mg N g^{-1} DW)				
			Leaf	Sheath or stem	Shoot	Tuber	Root
Wheat	aCO_2	Nitrate	39.87 ± 0.91	25.68 ± 1.70	35.88 ± 1.17		13.52 ± 0.80
		Urea	38.70 ± 1.45	22.86 ± 1.52	33.95 ± 1.41		13.37 ± 1.88
	eCO_2	Nitrate	30.84 ± 2.29	17.85 ± 1.85	26.62 ± 2.16		11.39 ± 1.42
		Urea	32.60 ± 1.40	18.77 ± 2.02	27.55 ± 1.81		11.77 ± 1.51
	**ANOVA**	**CO_2 (C)**	***P* < 0.001**	***P* = 0.006**	***P* = 0.001**		***P* = 0.226**
		**N form (N)**	***P* = 0.857**	***P* = 0.603**	***P* = 0.772**		***P* = 0.937**
		**C × N**	***P* = 0.375**	***P* = 0.314**	***P* = 0.410**		***P* = 0.860**
Rice	aCO_2	Nitrate	50.36 ± 0.62	24.04 ± 0.60	36.84 ± 0.71		15.10 ± 0.57
		Urea	51.67 ± 0.93	25.78 ± 0.44	38.76 ± 0.68		17.41 ± 0.88
	eCO_2	Nitrate	44.33 ± 0.90	21.60 ± 0.75	32.29 ± 0.52		14.82 ± 0.18
		Urea	42.49 ± 1.40	21.84 ± 0.92	31.37 ± 1.40		16.00 ± 0.11
	**ANOVA**	**CO_2 (C)**	***P* < 0.001**	***P* < 0.001**	***P* < 0.001**		***P* = 0.139**
		**N form (N)**	***P* = 0.799**	***P* = 0.799**	***P* = 0.581**		***P* = 0.007**
		**C × N**	***P* = 0.143**	***P* = 0.143**	***P* = 0.137**		***P* = 0.309**
Potato	aCO_2	Nitrate	64.48 ± 0.87	26.88 ± 2.07	52.27 ± 0.39	20.43 ± 1.70	21.39 ± 1.17
		Urea	68.88 ± 0.35	27.87 ± 1.81	56.44 ± 0.22	18.28 ± 0.83	21.66 ± 1.44
	eCO_2	Nitrate	61.94 ± 2.26	25.27 ± 1.77	50.81 ± 1.67	17.70 ± 1.37	21.99 ± 1.20
		Urea	62.59 ± 2.94	28.41 ± 1.40	52.90 ± 1.87	15.66 ± 0.76	21.07 ± 0.17
	**ANOVA**	**CO_2 (C)**	***P* = 0.040**	***P* = 0.770**	***P* = 0.073**	***P* = 0.050**	***P* = 0.992**
		**N form (N)**	***P* = 0.211**	***P* = 0.267**	***P* = 0.030**	***P* = 0.113**	***P* = 0.775**
		**C × N**	***P* = 0.345**	***P* = 0.556**	***P* = 0.430**	***P* = 0.965**	***P* = 0.599**
Guinea grass	aCO_2	Nitrate	23.21 ± 1.03	14.52 ± 0.55	18.72 ± 0.78		12.90 ± 0.65
		Urea	23.14 ± 0.57	15.42 ± 0.36	19.13 ± 0.22		13.70 ± 1.21
	eCO_2	Nitrate	19.90 ± 0.76	13.17 ± 0.51	16.55 ± 0.63		13.64 ± 0.99
		Urea	19.81 ± 0.64	13.19 ± 0.70	16.52 ± 0.50		14.19 ± 1.15
	**ANOVA**	**CO_2 (C)**	***P* = 0.001**	***P* = 0.006**	***P* = 0.001**		***P* = 0.558**
		**N form (N)**	***P* = 0.917**	***P* = 0.414**	***P* = 0.746**		***P* = 0.524**
		**C × N**	***P* = 0.990**	***P* = 0.429**	***P* = 0.705**		***P* = 0.905**
Amaranthus	aCO_2	Nitrate	41.65 ± 1.84	19.05 ± 0.77	33.78 ± 1.32		19.12 ± 0.46
		Urea	50.14 ± 3.24	25.11 ± 1.89	42.64 ± 2.77		20.81 ± 1.40
	eCO_2	Nitrate	40.19 ± 2.39	21.30 ± 2.49	33.27 ± 2.12		20.02 ± 1.87
		Urea	49.53 ± 0.93	24.43 ± 0.93	41.43 ± 0.56		22.09 ± 0.75
	**ANOVA**	**CO_2 (C)**	***P* = 0.656**	***P* = 0.648**	***P* = 0.648**		***P* = 0.400**
		**N form (N)**	***P* = 0.002**	***P* = 0.018**	***P* = 0.018**		***P* = 0.157**
		**C × N**	***P* = 0.854**	***P* = 0.399**	***P* = 0.399**		***P* = 0.884**

Each data is mean ± SE (n = 4).

Table S5. Nitrate accumulation in each organ in nitrate-fed or urea-fed plants in five species grown for 28 d in the chambers under ambient (aCO_2) or elevated (eCO_2) CO_2 treatments.

Species	CO_2	N-fed form	The percentage of nitrate-N in plant N (%)						$\delta^{15}N$ value in residue after nitrate extraction (‰)
			Leaf	Sheath or stem	Shoot	Tuber	Root	Whole plant	Whole plant
Wheat	aCO_2	Nitrate	6.19 ± 2.46	14.52 ± 3.77	8.05 ± 2.81		1.96 ± 0.23	7.11 ± 2.46	-1.88 ± 0.33
		Urea	2.57 ± 0.92	9.71 ± 2.68	4.13 ± 1.33		1.67 ± 0.41	3.73 ± 1.18	-3.60 ± 0.19
	eCO_2	Nitrate	1.02 ± 0.11	4.55 ± 1.30	1.84 ± 0.40		1.48 ± 0.44	1.82 ± 0.33	-0.10 ± 0.31
		Urea	0.37 ± 0.31	5.15 ± 0.96	1.59 ± 0.48		1.06 ± 0.24	1.50 ± 0.42	-3.49 ± 0.10
	**ANOVA**	**CO_2 (C)**	**$P = 0.016$**	**$P = 0.012$**	**$P = 0.017$**		**$P = 0.134$**	**$P = 0.019$**	**$P = 0.007$**
		**N form (N)**	**$P = 0.133$**	**$P = 0.408$**	**$P = 0.214$**		**$P = 0.317$**	**$P = 0.209$**	**$P < 0.001$**
		**C × N**	**$P = 0.284$**	**$P = 0.291$**	**$P = 0.270$**		**$P = 0.858$**	**$P = 0.292$**	**$P = 0.014$**
Rice	aCO_2	Nitrate	0.68 ± 0.11	10.22 ± 0.38	4.09 ± 0.20		1.35 ± 0.69	3.83 ± 0.18	2.42 ± 1.63
		Urea	0.72 ± 0.06	7.19 ± 0.18	2.96 ± 0.05		1.72 ± 0.63	2.85 ± 0.10	0.98 ±1.76
	eCO_2	Nitrate	0.97 ± 0.16	10.65 ± 0.26	4.65 ± 0.26		0.96 ± 0.16	4.25 ± 0.24	-1.03 ± 0.41
		Urea	1.02 ± 0.12	7.68 ± 0.74	3.62 ± 0.30		1.29 ± 0.39	3.35 ± 0.24	-1.67 ± 0.53
	**ANOVA**	**CO_2 (C)**	**$P = 0.027$**	**$P = 0.316$**	**$P = 0.018$**		**$P = 0.441$**	**$P = 0.040$**	**$P = 0.056$**
		**N form (N)**	**$P = 0.728$**	**$P < 0.001$**	**$P < 0.001$**		**$P = 0.513$**	**$P < 0.001$**	**$P = 0.485$**
		**C × N**	**$P = 0.975$**	**$P = 0.945$**	**$P = 0.817$**		**$P = 0.973$**	**$P = 0.835$**	**$P = 0.784$**
Potato	aCO_2	Nitrate	11.80 ± 1.68	47.60 ± 3.49	20.79 ± 1.64	3.23 ± 0.46	2.09 ± 0.61	17.09 ± 1.70	-2.58 ± 0.16
		Urea	3.05 ± 0.69	26.65 ± 3.52	7.47 ± 1.09	0.99 ± 0.80	2.17 ± 1.31	5.91 ± 0.84	-3.68 ± 0.15
	eCO_2	Nitrate	6.44 ± 1.35	37.74 ± 5.38	13.12 ± 2.35	2.72 ± 0.70	1.00 ± 0.61	10.09 ± 1.95	-1.41 ± 0.39
		Urea	2.09 ± 0.54	14.95 ± 4.38	4.45 ± 1.38	1.14 ± 0.47	0.56 ± 0.04	3.64 ± 1.05	-2.69 ± 0.03
	**ANOVA**	**CO_2 (C)**	**$P = 0.019$**	**$P = 0.026$**	**$P = 0.008$**	**$P = 0.944$**	**$P = 0.109$**	**$P = 0.008$**	**$P = 0.001$**
		**N form (N)**	**$P < 0.001$**	**$P < 0.001$**	**$P < 0.001$**	**$P = 0.015$**	**$P = 0.821$**	**$P < 0.001$**	**$P < 0.001$**
		**C × N**	**$P = 0.083$**	**$P = 0.833$**	**$P = 0.193$**	**$P = 0.470$**	**$P = 0.745$**	**$P = 0.131$**	**$P = 0.732$**
Guinea grass	aCO_2	Nitrate	0.80 ± 0.29	8.08 ± 2.18	3.88 ± 1.12		0.90 ± 0.30	3.46 ± 0.94	1.45 ± 0.13
		Urea	0.42 ± 0.16	9.41 ± 1.27	4.39 ± 0.66		0.51 ± 0.28	3.85 ± 0.54	-2.05 ± 0.16
	eCO_2	Nitrate	0.60 ± 0.14	2.76 ± 0.49	1.46 ± 0.17		0.97 ± 0.23	1.38 ± 0.18	1.55 ± 0.13
		Urea	1.08 ± 0.52	2.00 ± 0.24	1.46 ± 0.39		0.50 ± 0.21	1.31 ± 0.33	-1.74 ± 0.13
	**ANOVA**	**CO_2 (C)**	**$P = 0.472$**	**$P < 0.001$**	**$P = 0.002$**		**$P = 0.917$**	**$P = 0.002$**	**$P = 0.221$**
		**N form (N)**	**$P = 0.883$**	**$P = 0.832$**	**$P = 0.719$**		**$P = 0.121$**	**$P = 0.779$**	**$P < 0.001$**
		**C × N**	**$P = 0.201$**	**$P = 0.435$**	**$P = 0.713$**		**$P = 0.864$**	**$P = 0.696$**	**$P = 0.533$**
Amaranthus	aCO_2	Nitrate	2.15 ± 0.26	16.67 ± 2.88	5.40 ± 0.78		1.72 ± 0.48	5.08 ± 0.75	0.05 ± 0.16
		Urea	2.68 ± 0.97	21.54 ± 6.78	6.68 ± 2.06		1.32 ± 0.46	6.31 ± 2.00	-2.63 ± 0.50
	eCO_2	Nitrate	4.38 ± 1.14	19.43 ± 6.07	8.84 ± 3.04		2.08 ± 1.37	8.30 ± 2.81	0.25 ± 0.39
		Urea	2.00 ± 0.27	17.72 ± 2.63	5.36 ± 0.56		0.38 ± 0.08	4.93 ± 0.51	-3.24 ± 0.26
	**ANOVA**	**CO_2 (C)**	**$P = 0.335$**	**$P = 0.916$**	**$P = 0.586$**		**$P = 0.710$**	**$P = 0.615$**	**$P = 0.611$**
		**N form (N)**	**$P = 0.251$**	**$P = 0.755$**	**$P = 0.571$**		**$P = 0.195$**	**$P = 0.560$**	**$P < 0.001$**
		**C × N**	**$P = 0.083$**	**$P = 0.519$**	**$P = 0.234$**		**$P = 0.412$**	**$P = 0.222$**	**$P = 0.336$**

Each data is mean ± SE (n = 4).

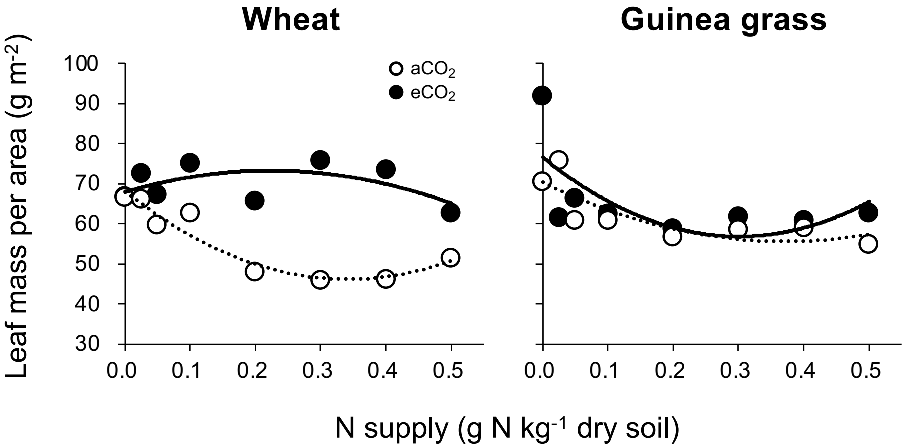

Figure S2. Changes in leaf mass per area of wheat or guinea grass according to N supply grown for 28 d in the chambers under ambient (aCO_2) or elevated (eCO_2) CO_2 treatments.

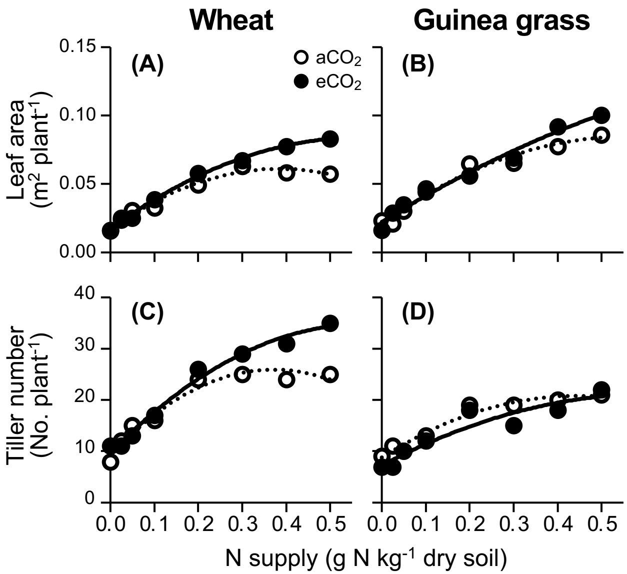

Figure S3. Changes in leaf area (A, B) and the number of tillers (C, D) of wheat or guinea grass according to N supply grown for 28 d in the chambers under ambient (aCO_2) or elevated (eCO_2) CO_2 treatments.
